# Spino-Plastic Surgery: Addressing Spinal Tumors with New Techniques

**DOI:** 10.3390/cancers16234088

**Published:** 2024-12-06

**Authors:** Casey Martinez, Isra Abdulwadood, Sebastian Winocour, Alexander E. Ropper, Marco Innocenti, Michael Bohl, Maziyar Kalani, Edward M. Reece

**Affiliations:** 1 Mayo Clinic Alix School of Medicine, Phoenix, AZ 85054, USA; martinez.casey@mayo.edu (C.M.); abdulwadood.isra@mayo.edu (I.A.); 2 Department of Plastic Surgery, Baylor College of Medicine, Houston, TX 77030, USA; sebastian.winocour@bcm.edu; 3 Department of Neurosurgery, Baylor College of Medicine, Houston, TX 77030, USA; alexander.ropper@bcm.edu; 4 The Rizzoli Orthopaedics Institute, 40136 Bologna, Italy; marco.innocenti@ior.it; 5 Carolina Neurosurgery & Spine Associates, Charlotte, NC 28204, USA; michael.bohl@cnsa.com; 6 Department of Neurosurgery, Mayo Clinic, Phoenix, AZ 85054, USA; kalani.mazi@mayo.edu; 7 Department of Surgery, Division of Plastic and Reconstructive Surgery, Mayo Clinic, Phoenix, AZ 85054, USA

**Keywords:** spino-plastic surgery, surgical oncology, spinal surgery, complex reconstruction, vascularized bone graft

## Abstract

**Background/Objectives:** Spino-plastic surgery describes a specialized, multidisciplinary approach to addressing various spinal pathologies. The field is the innovative product of a multidisciplinary collaboration between plastic and reconstructive, orthopedic, and neurosurgery. Over the last few decades, this collaboration has borne promising surgical techniques and treatment plans geared toward restoring form, function, and aesthetics in patients with a variety of spinal conditions, including failed fusions, pseudoarthrosis, and the need for oncologic reconstruction. This paper explores the application of spino-plastic surgery in the context of post-sarcoma resection reconstructions, focusing on the efficacy in addressing the unique challenges posed by extensive tissue loss and structural deformities. **Methods:** Our study reviews a series of cases wherein spino-plastic techniques were utilized in patients with sarcomas of the spine and adjacent structures. We also discuss the technical considerations, including preoperative planning, intraoperative challenges, and overall patient care, that are crucial for the success of spino-plastic procedures. **Results:** The outcomes demonstrate significant improvements in patient mobility, pain reduction, and overall quality of life. Most notably, spino-plastic surgical techniques help facilitate the restoration of functional anatomy by leveraging vascularized bone grafts and muscle flaps, thereby enhancing long-term stability and reducing the risk of complications such as nonunion or infection. **Conclusions:** Spino-plastic collaboration represents a pivotal advancement in oncologic treatment, spinal care, and reconstructive surgery, offering new hope for patients undergoing post-sarcoma reconstruction. Further research and refinement of the techniques will only expand their application and improve outcomes for a broader range of patients in the future.

## Simple Summary

The oncologic resection of spinal tumors often necessitates complex spinal reconstruction. Spino-plastics is a field that utilizes vascularize bone grafts (VBGs), which are pedicled on muscle, to augment spinal fusion. There are many variables that drive the approach to oncologic spinal reconstruction, including the complications encountered during tumor extirpation, the mechanics and stability of the affected spinal segment, the dimensions, and the level of the defect. In this article, we discuss the spino-plastics framework for reconstruction, describing the benefits of VBGs and the various options for harvest. Additionally, we describe the future of spino-plastics and various innovations in surgical oncology that are contributing to improved utilization of VBGs following sarcoma resection.

## 1. Introduction

Spinal osteosarcomas comprise up to 15% of all primary spine tumors [1] and 0.6–3.2% of all sarcomas are located in the spine [2]. While rare, these tumors present significant clinical challenges due to their aggressive nature and propensity to present as high-grade malignancies of the thoracolumbar spinal region [2]. There are a wide range of management options, including chemotherapeutic, radiotherapeutic, surgical, and combination treatment regimens that are indicated based upon oncologic staging and shared decision making between patient and provider teams [3]. From a surgical standpoint, interventions span from palliative stabilization or minimally invasive approaches to the most invasive of options, such as en bloc spondylectomy, or the removal of the entire vertebral body and lamina as one unit [4,5,6]. The literature suggests that long-term survival is directly correlated with the extent of the resection in the setting of primary spinal malignancies [7], and aggressive pathology often warrants a more invasive approach when options are limited for curative treatment.

The emerging field of spino-plastic surgery has introduced innovative strategies that address challenging spinal reconstruction, particularly via the utilization of vascularized bone grafts (VBGs). Supplied by Sharpey’s fibers and pedicled on muscle to augment fusion, VBGs rely on the osteogenic, osteoinductive, and osteoconductive properties of vascularized bone, creating an ideal environment for wound healing and fusion [8,9]. Through this multidisciplinary collaboration between neurosurgery, orthopedics, and plastic and reconstructive surgery (PRS), spino-plastic surgery can tackle challenging problems and offer promising solutions that enhance postoperative outcomes and improve long-term quality of life for patients.

## 2. Oncologic Spinal Reconstruction

Vertebral body defects requiring surgical intervention can arise from a variety of etiologies, including malignancy, trauma, congenital deformity, degenerative conditions, and infection [10,11]. In surgical oncology, tumor characteristics often dictate the complexity of resection, necessitating a rehabilitative approach tailored to each case.

Vertebral augmentation procedures fall into two main categories: vertebroplasty and kyphoplasty. Both techniques aim to alleviate pain and enhance vertebral stability by injecting synthetic materials, such as polymethyl methacrylate (PMMA) bone cement, into the vertebral body [10]. Kyphoplasty utilizes an inflatable balloon catheter to create a cavity within the vertebra before cement is introduced, making it particularly beneficial for cases requiring height restoration in collapsed vertebrae or the correction of spinal kyphosis [10]. Synthetic materials offer structural support and simplify procedures by eliminating the need for autologous tissue harvesting, thereby reducing operative time, complexity, and potentially leading to fewer immediate complications and shorter hospital stays.

In cases with minor resection and a lower risk of postoperative instability, minimally invasive techniques like vertebroplasty and kyphoplasty may be sufficient. However, these procedures, which inject synthetic bone cement into the affected vertebral trabeculae, are often inadequate for more complex cases [10]. Unlike vascularized bone grafts (VBGs), which provide osteogenic, osteoinductive, and osteoconductive support, synthetic materials lack the biological activity essential for bone integration and healing. This limitation can lead to higher rejection rates, increased infection risks, and inadequate fusion, particularly in patients undergoing adjuvant therapies, where graft viability is critical. Additionally, oncologic patients may experience reduced compatibility with synthetic materials, leading to more frequent complications or the need for revision surgeries.

Minimally invasive procedures also carry inherent risks, including cement extravasation and adjacent segment compression fractures. To mitigate these complications, guidelines recommend limiting cement to three or four vertebral levels per session and treating complex fractures across multiple sessions [10]. Vertebroplasty and kyphoplasty are contraindicated in specific situations, including moderate to severe deformities, vertebra plana, unstable fractures with compromised ligamentous integrity, spinal cord compression, cement allergies, and chronic fractures [10,11,12].

In more extensive cases, wide local resection offers the highest likelihood of oncologic cure but comes with substantial risks, such as injury to major vessels during anterior vertebral exposure, spinal cord vascular compromise, excessive epidural or venous bleeding, mechanical stress on neural structures, and spinal column shortening [4,6]. Effective care for this complex patient population often requires innovative reconstructive approaches. The extent and location of the defect following tumor removal largely determines the appropriate reconstructive method. Adequate arthrodesis, preserving the anatomical length and curvature of the affected and adjacent spinal segments, is essential for favorable outcomes. Without reliable reconstruction, patients face risks of spinal instability, pseudoarthrosis, and neurovascular compromise, potentially leading to significant morbidity and mortality.

While synthetic grafts and current surgical techniques, whether minimally invasive or through wide resection, offer practical solutions in select cases, the biological and functional benefits of VBGs make them a preferred option for patients needing robust, vascularized support in complex spinal reconstructions.

## 3. The Role of VBGs in Spinal Reconstruction

Plastic surgeons have long been entrusted with the role of wound care experts, taking on a reactive role in the postoperative complications of spine surgery [13,14]. VBGs are not a new concept as they have been described in the spine literature dating back to 1982 [15]. However, the re-evaluation of VBGs through the spino-plastic lens has expanded the reach and application of VBGs, catapulting PRS to the forefront of innovation in spinal surgery.

The main concept remains simple: vascularized tissue leads to improved healing. Paraspinal muscle flaps have been utilized for many years for more effective spinal hardware coverage, lower incidence of reoperation, and ease of postoperative hardware retrieval, if necessary [16]. The literature demonstrates that muscle flaps alone can significantly improve the ability to heal, decreasing complex spinal wound complication rates through a reduction in both dead space and donor site morbidity, with simultaneously improved vascularity of the wound bed [16]. Moreover, Skochdople et al. adapted the reconstruction algorithm, incorporating VBGs into six distinct levels: allograft, bony substitute, autograft, N-VBGs, VBGs, pedicled vascularized bone flap, and finally, free bone flaps [9,17]. Chimeric flaps, of which VBGs are one type, involve the combination of different tissue types or independent perforasomes, and are employed to address the reconstruction of extensive bony and soft tissue defects [18,19,20].

Sharpey’s fibers serve as the link from muscle to bone, providing vascularity from small unnamed periosteal feeding vessels to the Haversian canals [9]. By harnessing the orthobiological mechanisms of healing, introducing osteocytes directly to the wound bed, VBGs are a single-stage operation that offer a comprehensive solution without significantly increasing operative time or requiring the microsurgical skill associated with free flap procedures. Standard arthrodesis equipment is sufficient for VBG harvest and placement, as there is no required preparation of donor or recipient vessels. Compared to a free fibula flap, one of the mainstay procedures for vascularized grafting, pedicled grafts do not incur the same donor site morbidity, increased costs, or prolonged patient recovery time [21,22].

## 4. VBG Anatomy and Dissection

There are six main types of VBGs described in the literature, which include harvesting bone from the iliac crest (IC-VBG) [23,24,25], ribs (R-VBG) [26,27], scapula (S-VBG) [28], occiput (O-VBG) [29,30,31], spinous processes (SP-VBG) [32], and clavicle (C-VBG) [17]. Each of these graft types have maximal arc of rotation based upon the anatomic location and length of the muscular pedicle, as well as variability in vascular contribution or necessity to preserve key structures during dissection. It is also important to consider the native structure and biomechanical stress of the VBG donor site, adhering to the maximal recommended graft length, so as not to inadvertently introduce instability elsewhere. Deciding which VBG to utilize largely depends on the status of the spinal defect status post-spinal tumor resection. After the instrumentation and fusion is completed in the typical fashion, the spino-plastics team may then make the determination of which segment is most critical for successful fusion. Once the end target for fixation is decided, the harvest of the most appropriate type of VBG is begun. Prior to the hardware fixation of the VBG to the decorticated spinal surface at the target site, active bleeding at the medullary surface of the VBG can often be noted on direct visualization. For further confirmation of graft viability, indocyanine green may be administered to the patient intravenously and traced through the bleeding edge [23].

**Figure 1:**
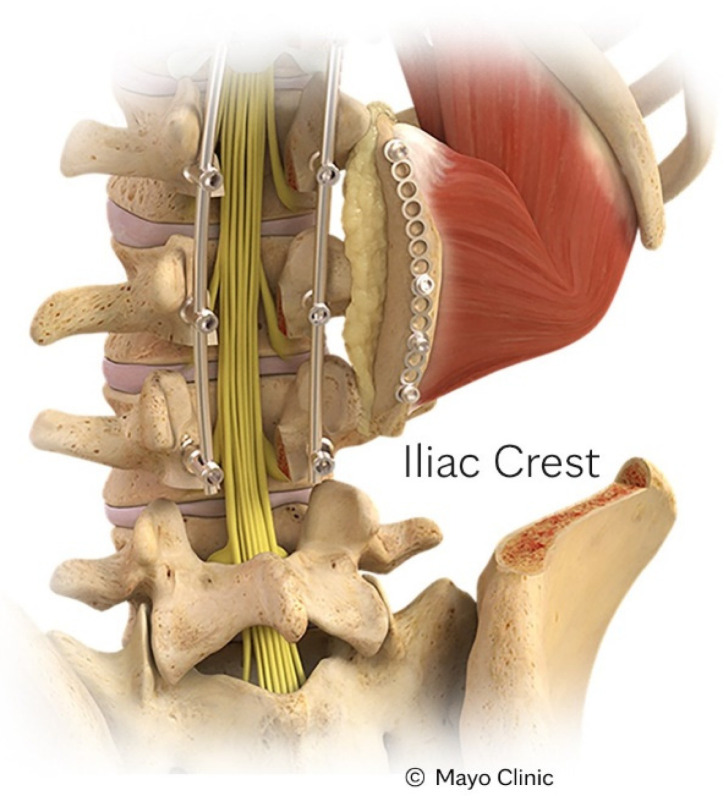
Depiction of an IC-VBG. Reproduced with the permission from Mayo Clinic.

The IC-VBG [23,24,25,26,27] was the first option described in literature, and is the most widely utilized of the VBGs. This graft type, shown in Figure 1, is often used for coverage of the distal thoracic and lumbar spine, specifically T12 to sacrum. Pedicled on the quadratus lumborum, there is multifactorial contribution to Sharpey’s fibers. As with all VBGs, it is important to perform a subperiosteal dissection to preserve pertinent neurovascular structures. In this case, the surgeon must take care to avoid disruption of the cluneal nerve. Up to 10 cm of tricortical cancellous bone can be harvested to support larger spinal defects, or those at greater risk of fusion failure.

**Figure 2:**
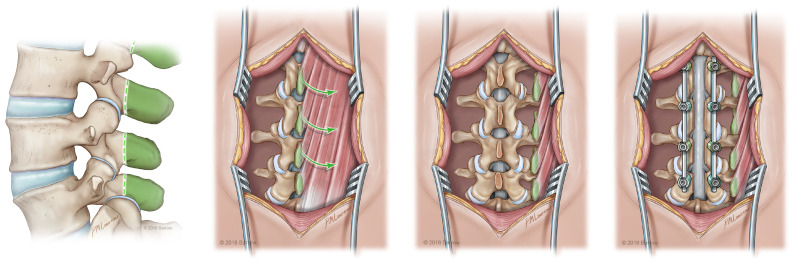
Depiction of a spinous process VBG harvest to fixation with spinal stabilization from left to right, reproduced with the permission of the Barrow Neurological Institute, Phoenix, AZ, USA.

In terms of coverage of levels within the thoracolumbar region, the SP-VBG is an excellent option, depicted in Figure 2 [32]. Paraspinal perforators supply the paraspinal muscles that become the pedicle for this graft. The exposure for this donor site has already been achieved during the spinal fusion itself. After graft placement, wire cables or resorbable sutures are used to stabilize the posterolateral space prior to fascial closure, minimizing the risk of graft extrusion.

**Figure 3:**
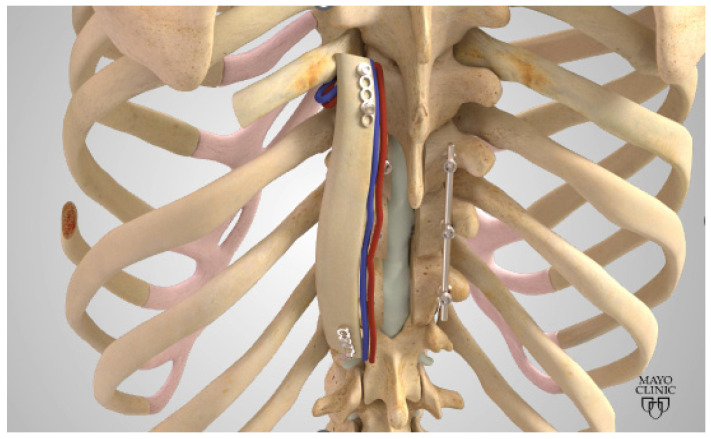
Depiction of a Rib VBG. Reproduced with the permission from Mayo Clinic.

Similar to the SP-VBG, the arc of rotation for the R-VBG is C6-L5 when using the 8th rib [26,27]. While this rib level is typical, other ribs may be considered based upon location and curvature to best match the kyphosis or lordosis of the natural spine. The R-VBG is attached to the subcostal muscle, supplied by the subcostal artery, as shown in Figure 3. When harvesting this type of graft, the patient is at greatest risk of pneumothorax. With careful blunt dissection, the lung pleura and intercostal nerve traversing the inferior aspect of the rib can be averted. A bubble study is easily performed to confirm the integrity of the pleura and allow for quick identification of perforation in any areas of concern. 

**Figure 4:**
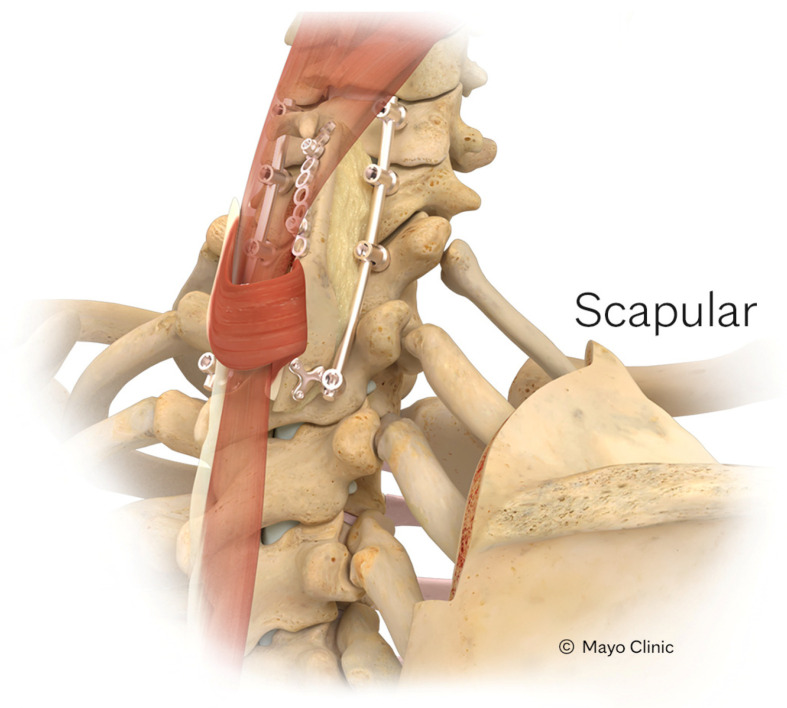
Depiction of a scapular VBG. Reproduced with the permission from Mayo Clinic.

**Figure 5:**
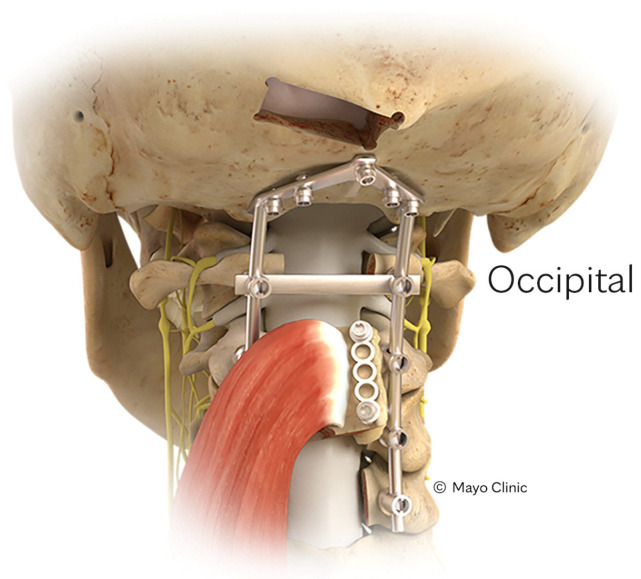
Depiction of an occipital VBG. Reproduced with the permission from Mayo Clinic.

**Figure 6:**
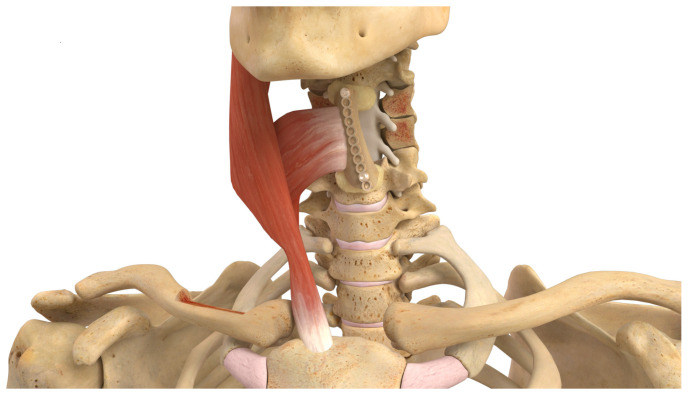
Depiction of a clavicular VBG. Reproduced with the permission from Mayo Clinic.

Superiorly along the spine, cervical spinal defects might be reached by the S-VBG [28], O-VBG [29,30,31], and C-VBGs [17]. Pedicled on the rhomboid minor or trapezius, the S-VBG’s blood supply originates in a multifactorial manner and may span from the occiput down to T8, as seen in Figure 4. Care must be taken to preserve the descending branch of the transverse cervical artery, which may be encountered during dissection of the medial border of the scapula. The O-VBG, depicted in Figure 5, requires meticulous dissection, with the inferior border of the occiput being the foramen magnum. The deep cervical artery originates from the costocervical trunk within the semispinalis capitis muscle, which is a Mathes-Nahai type II flap, reducing the likelihood of vascular kinking and flap compromise upon extension. With a slightly broader reach from C2 to T2, the C-VBG, as seen in Figure 6, is based on the clavicular head of the sternocleidomastoid muscle, which is supplied by the branches of the occipital and superior thyroid arteries. Caution while dissecting in the anterior cervical triangle is important to avoid important neurovascular structures such as the carotid artery, jugular vein, and several nerves inclusive of the hypoglossal, accessory, vagus, glossopharyngeal, and facial nerves. Cosmesis and muscular function may be best preserved by taking only the superior medial 2/3 of the clavicle and keeping the bony attachments to the inferior pectoralis and superior sternocleidomastoid muscles.

## 5. Outcomes and Complications

At present, the long-term outcomes and potential complications associated with these interventions remain under investigation. As our institutional experience grows and the long-term patient cohort reaches a sufficient size, we hope to perform reliable statistical analysis. It is of note that many patients with extensive sarcoma resection undergo subsequent chemotherapy and radiation. Preoperative chemotherapy or neoadjuvant treatments may be advised in the case of high-grade or pathologically distinct tumors [33]. This presents additional complexity in attaining successful fusion, stability, and overall wound healing.

Large non-vascularized allografts have been shown to have high complication rates of non-unions, infections, and fractures [34]. VBGs circumvent and combat these complications by providing vascularity for direct bone healing at the defect site. In the plastic surgery literature, it has been established that the incorporation of well-vascularized, healthy tissue results in more favorable outcomes, with a decreased risk of wound breakdown and local necrosis. This highlights the critical role of vascularized bone grafts (VBGs) in managing patients at heightened risk for impaired wound healing, particularly those undergoing reconstruction following sarcoma extirpation. Thus far, patients who have undergone spino-plastic procedures have fared well in the short-term postoperative period. Our anecdotal experience has shown faster fusion rates with VBG compared to those who have not undergone spinoplastic procedures in addition to the traditional fusion technique, with or without non-vascularized grafting.

VBG survivability has yet to be explored extensively due to the current lack of longitudinal data. However, published case reports detail successful IC-VBG incorporation on long-term postoperative imaging, even in the setting of high-risk patients such as those with lumbar osteomyelitis and discitis and many prior failed fusions [24,35]. Pinosolle et al. reported a 100% bony union rate for eight patients with humeral defects undergoing S-VBGs, with a time to complete union of 4–6 months [36]. Wilden et al. also found a 100% fusion rate with very low overall morbidity for 18 R-VBG cases, which took an average of 6.8 months to complete union [37]. Other smaller case series show 87.5% [38,39] and 93% [40] fusion rates, with averages of 6 months or less to bony union [38,40]. Complications aside from nonunion included low rates of hematoma [36], the need for further surgery [36], proximal fixation loosening without functional impact [40], and bony hypertrophy [37,40,41]. In one case series of 14 IC-VBGs with concurrent lumbar spine fusions, there were no reported spigelian hernias or any significant donor site pain [23].

Spinal instrumentation and fusion are associated with blood loss that often necessitates transfusions in the perioperative setting, due to the high vascularity and longer operative times required for extensive work on the spine. Blood loss ranges from 650 to 2800 cc for one adult spine fusion case [42]. Further studies are required to delineate the specific blood loss associated with only the VBG harvest and fixation within the larger context of the surgery. Harvest and fixation of the VBG does not take significant operative time. The exact duration depends upon the nature of the graft, including aspects such as the size and location of the donor site, as well as whether the procedure is unilateral or bilateral. Analysis of the exact operative times is part of the next steps we are taking while compiling outcome data.

## 6. Advantages of Spino-Plastics in Complex Cases

The multidisciplinary approach of spino-plastic surgery, particularly the application of VBGs, represents a significant advancement in the management of complex spine cases. Traditional spinal reconstruction techniques rely on non-vascularized bone grafts (N-VBGs) or hardware which can be limiting due to the risk of complications associated with foreign bodies such as graft or hardware failure, infection, the need for revision surgeries, chronic pain, and more [43,44]. VBGs, alternatively, offer unique advantages. By retaining a reliable and rather robust blood supply, instead of creeping substitution as is the case for N-VBGs, VBGs are more likely to survive and integrate into the surrounding tissue, reducing the risks associated with foreign bodies and graft failure.

The primary advantage of VBGs in spino-plastic applications is the graft’s intrinsic capacity for osteogenesis, osteoinduction, and osteoconduction. The biologic ability to generate new bone, induce bone growth, and provide a scaffold for future bone formation is particularly beneficial in cases of extensive bone loss or when the surrounding tissue is compromised. This is the case for many oncology patients who have undergone radiation therapy or in those who have failed previous fusions.

Furthermore, the integration of VBGs into complex spinal reconstructions leverages the expertise of plastic surgeons in a proactive manner. Instead of joining the team in the context of wound closure or postoperative wound care, plastic surgeons are involved in the creation of custom-designed grafts that meet the unique needs of each patient following sarcoma resection, ultimately reducing the individual and systemic burden of spinal care. This approach can enhance the durability of spinal reconstructions, ensuring spinal stability and lowering the risk of neurologic injury, hardware failure, and the need for future revisions. Patients can benefit from improved soft tissue coverage, enhancing overall aesthetic and functional outcomes.

## 7. Limitations

While VBGs offer significant advantages in spinal reconstruction, including enhanced graft viability and reduced complication rates, their use is not without limitations. A key challenge lies in the specialized expertise required for these procedures, which necessitates collaboration between spine and plastic surgeons. However, access to plastic surgeons trained in spino-plastic techniques is not universally available as these techniques are still relatively novel and can involve longer operative times, increased technical complexity, and a steep learning curve, which may restrict their applicability to even specialized centers. These factors present logistical and practical barriers to the widespread adoption of VBG techniques in spinal oncology and other complex spinal pathologies.

Furthermore, a limitation exists within early plastic surgery training itself. Traditional plastic surgery residencies and fellowships focus on general reconstructive principles and microvascular techniques but may not provide specific training in spino-plastic approaches or spine-centered vascularized bone grafts. While VBGs build upon the core principles of plastic surgery, surgeons may have limited exposure to the specific nuances involved in applying these techniques within the context of spinal reconstruction. Addressing this gap through dedicated spino-plastic training modules or collaborative spine-plastics fellowships could help bridge this expertise gap and broaden the availability of these reconstructive options.

Finally, the authors of this paper recognize that many healthcare institutions, particularly those in rural or underserved areas, may lack plastic surgeons entirely, further limiting patient access to the full benefits of VBGs. Encouraging the widespread and early adoption of collaboration between spine surgeons and plastic surgeons can enhance spine surgeons’ familiarity with the reconstructive principles that contribute to improved outcomes. Thus, the involvement of a plastic surgeon is strongly encouraged. The complexities of vascularized bone grafts and soft tissue reconstruction require specialized expertise that plastic surgeons distinctly bring to the multidisciplinary team, particularly in managing intricate wound closures and ensuring optimal vascularity for graft viability. Integrating plastic surgeons not only enhances patient outcomes but also provides a more robust surgical approach, reducing complications and the need for revisions. Thus, we advocate for collaborative models that leverage the strengths of both plastic and spine surgery specialties to maximize patient benefit in complex spinal reconstructions.

## 8. Innovations and the Future

PRS and spinal surgery both innately require an innovative and multidisciplinary form of practice. The combination of an increasingly high-risk, aging patient population, and growing global disease burden add a degree of difficulty to the treatment of complex spine reconstruction. Spino-plastic surgery is a collaborative, precise, and creative framework that was built to enhance the healing, integration, and durability in spinal fusion amidst challenging circumstances. The body of evidence for the utilization of VBGs in oncologic spinal reconstruction is growing. Looking to the future, there is an opportunity to hone surgical techniques in the harvest and fixation of VBGs.

In response to the rise of spino-plastic surgery, many institutions, nationally and globally, are establishing multidisciplinary clinics that mirror the collaborative model seen in craniofacial surgery [45]. These clinics provide spine and plastic surgeons to interface directly with patients, ensuring a comprehensive and long-term approach to pre-, intra-, and postoperative care. By coordinating efforts and building cross-specialty connections, spino-plastic clinics can facilitate more tailored treatment plans, enhance communication, and optimize functional and aesthetic options, improving the quality of care and positioning spino-plastic techniques as the standard of practice for complex spine cases [46].

Following the trends of other surgical subspecialties, artificial intelligence (AI) has already demonstrated the ability to improve surgical planning and intraoperative precision. Custom-made models and implants are only a part of data-driven solutions that are improving in quality since the broader introduction and application of AI in spinal surgery. As time goes on, it is clear that computer scientists, bioinformaticians, and data engineers will play an integral role in the future of spine surgery, processing large amounts of data and sophisticated information that is specific to each unique patient, which can provide a more detailed and scoping understanding of spinal reconstruction for research and clinical care [47].

AI, and machine learning (ML) in particular, has the potential to revolutionize several aspects of spino-plastics. ML predictive modeling can generate data points based on realistic synthetic populations that give surgeons the ability to better plan for possible outcomes and complications of various therapeutic approaches to patient care [48]. The immense predictive power of ML in surgery is evidenced by the ability to accurately predict postoperative complications, the use of pain medications, and other outcomes such as quality of life metrics [49]. As the number of spino-plastics cases continues to rise, there will be more data available for integration into AI algorithms. Optimizing patient selection criteria and indications for VBGs will be an integral part of advancing the field. ML is well positioned to assist in this task by effectively screening patients to determine those who are at high-risk of pseudoarthrosis and those who might reap the most benefit from the proactive employment of a VBG.

The surgeon’s preoperative toolkit has already been substantially advanced through augmented reality (AR) and virtual reality (VR), offering benefits to both seasoned surgeons and trainees alike. This technology permits simulated operative experiences, easier visualization of patient anatomy with the capability to combine and overlay aspects of various imaging studies, simpler patient education, and remote access that solves the problem of geographic barriers to education and patient care [50]. Virtual surgical planning has proven to be accurate and allows for improved symmetry and results, especially in cases where aesthetics are of paramount importance, such as orthognathic surgery [51].

Intraoperatively, AR is useful when in close proximity to several critical structures or at times when exposure is limited. When native anatomy is distorted by tumor bulk or any prior surgery to the affected site, as is the case in revision arthrodesis, the identification of key anatomic structures is made even more challenging. AR is already being used by spine surgeons to reduce the margin of error and ensure the safety of foraminotomies, bone biopsies, pedicle screw placement, percutaneous intervention and osteotomies [50]. Osteotomy placement can be virtually mapped based upon the dissection plane and volume to guide microscopic resection intraoperatively [52]. There is ample opportunity for spino-plastics to evolve with the technological landscape. With improved technology comes better analysis of results, operative performance, and overall patient outcomes.

## 9. Conclusions

Spino-plastic surgery represents a paradigm shift in the treatment of complex spinal cases, especially involving oncologic resection. By integrating the core principles of the reconstructive ladder and microsurgery with spinal surgery, this multidisciplinary approach enhances the ability to achieve durable, functional, and aesthetically-pleasing outcomes. VBGs have emerged as a critical tool in this domain, offering superior healing potential, reduced complication rates, and improved structural integrity compared to traditional approaches. Looking ahead, the future of spino-plastics is bright with ongoing advancements in surgical techniques and the integration of cutting-edge technologies such as AI and AR. We hope these technologies point to a future of refined patient selection, surgical planning, and intraoperative precision, elevating spino-plastics as the gold standard of spinal care in the future. As the field continues to evolve, collaboration across specialties will remain key, ensuring patients receive the highest standard of care while also reducing the global burden of disease.

## Data Availability

Not applicable.
